# *In Silico* Evaluation of Variant Calling Methods for Bacterial Whole-Genome Sequencing Assays

**DOI:** 10.1128/jcm.01842-22

**Published:** 2023-07-10

**Authors:** Yee Mey Seah, Mary K. Stewart, Daniel Hoogestraat, Molly Ryder, Brad T. Cookson, Stephen J. Salipante, Noah G. Hoffman

**Affiliations:** a Department of Laboratory Medicine and Pathology, University of Washington Medical Center, Seattle, Washington, USA; b Department of Microbiology, University of Washington, Seattle, Washington, USA; National Institute of Allergy and Infectious Diseases

**Keywords:** bioinformatics, whole-genome sequencing, bacterial genomes, next generation sequencing, computer simulation, DNA mutational analysis

## Abstract

Identification and analysis of clinically relevant strains of bacteria increasingly relies on whole-genome sequencing. The downstream bioinformatics steps necessary for calling variants from short-read sequences are well-established but seldom validated against haploid genomes. We devised an *in silico* workflow to introduce single nucleotide polymorphisms (SNP) and indels into bacterial reference genomes, and computationally generate sequencing reads based on the mutated genomes. We then applied the method to Mycobacterium tuberculosis H37Rv, Staphylococcus aureus NCTC 8325, and Klebsiella pneumoniae HS11286, and used the synthetic reads as truth sets for evaluating several popular variant callers. Insertions proved especially challenging for most variant callers to correctly identify, relative to deletions and single nucleotide polymorphisms. With adequate read depth, however, variant callers that use high quality soft-clipped reads and base mismatches to perform local realignment consistently had the highest precision and recall in identifying insertions and deletions ranging from1 to 50 bp. The remaining variant callers had lower recall values associated with identification of insertions greater than 20 bp.

## INTRODUCTION

Whole-genome sequencing and identification of bacteria can be immensely useful in tracking the transmission and evolution of pathogens during outbreaks, as well as in predicting clinically relevant phenotypes such as antimicrobial resistance. Clinical bioinformatics pipelines typically take advantage of known reference genomes that are medically important and rely on the accuracy of variant calling algorithms to identify pathogenic species and strains or to detect clinically relevant genotypes. Therefore, the accuracy of variant calling is critical in informing clinical decisions; however, the performance of many variant callers used in clinical microbiology workflows have primarily been developed for and evaluated against human reference genomes. Truth sets of bacterial species variants are limited (1). In addition, the conclusions of variant caller validation against linear and diploid reference genomes do not always apply to variant calling in haploid and circular bacterial genomes. For example, genotyping alleles relies on setting the appropriate minimum threshold for variant allele frequencies (VAF), and the VAF threshold for genotyping homozygous versus heterozygous alleles in diploid genomes necessarily differs from the minimum threshold for identifying alternate alleles in haploid genomes; variant callers that do not account for this may not be appropriate for bacterial variant calling.

Given these issues, various efforts to develop recommendations for benchmarking variant caller performance against bacterial reference genomes have been made, although these analyses have focused on the identification of single nucleotide variants exclusively (1–3). Bush et al. (1) evaluated bacterial single nucleotide polymorphisms (SNP)-calling pipelines using real and simulated reads of several species of *Enterobacteriaceae* and found that reference genome selection significantly impacts the performance of variant-calling pipelines, especially for highly recombinogenic bacterial species. This corresponds to the findings by Pightling et al. (4), which concluded that selection of both reference genomes and short read aligners affect variant calling in the clonal Listeria monocytogenes species.

One reason benchmarking analyses are restricted to evaluating SNP calls is the diversity of indel-calling algorithms (1), which prohibits direct performance comparisons. Indel-containing reads are challenging to map to unique genomic locations; both insertions and deletions can generate alternative haplotypes that correspond to multiple loci on reference haplotypes (5). Independent mapping of individual fragments by read mappers is also more likely to tolerate mismatches than gapped alignments (5). Therefore, in addition to alignment-based methods, other algorithms have been developed specifically for indel-calling, including split-read mapping, paired-end read mapping, and haplotype-based methods (6). Alignment-based indel callers rely on different models to distinguish true indels from alignment errors. Meanwhile, both split-read and paired-end read mapping methods use paired reads to identify discordant pairs for *de novo* assembly, and compare expected to actual mapped distance between pairs, respectively (6). Finally, haplotype-based methods identify active regions with evidence of indels relative to a reference sequence, followed by reassembly of the active regions to generate possible haplotypes from the reads; indels are then called based on the posterior probabilities of reads realigned to the possible haplotypes.

We expand on previous efforts to validate bacterial variant-calling by developing a toolkit for introducing synthetic variants into a reference genome and measuring the precision and recall of different callers in identifying not only SNPs, but also insertions and deletions. This validation is motivated by an effort to develop a clinical assay to detect antimicrobial resistance-associated mutations in M. tuberculosis. To determine the generalizability of our methods, we further extended the validation of these tools to other bacterial species of varying genome sizes and GC content: Mycobacterium tuberculosis H37Rv (4.4 Mbp, GC% 65.6), Klebsiella pneumoniae HS11286 (5.3 Mbp, GC% 57.5), and Staphylococcus aureus NCTC 8325 (2.8 Mbp, GC% 32.9). We then used the *in silico* altered genomes to test the performance of eight variant callers: bcftools, DeepVariant, DiscoSNP, FreeBayes, GATK HaplotypeCaller, Lancet, Octopus, and VarDict (Java implementation). We chose these variant callers because they are widely used, have good documentation, and appear to be actively maintained within the last 5 years, all of which are important criteria for software deployed in clinical pipelines.

Algorithms for identifying sequence variants from short reads can be broadly categorized into reference-based methods that rely on mapping reads onto a curated reference genome, and newer reference-free methods that construct deBruijn graphs of k-mers; some methods are a hybrid of both. Because variants of clinical significance are typically annotated relative to a reference strain, all the variant callers we tested are reference-based methods, or in the case of DiscoSNP, implemented in reference-based mode (Table 1).

**TABLE 1:** Summary of tools

Variant callers	Methods		Reference
		*Ploidy = 1?*	
bcftools v1.13	Allele frequency estimation based on mapped reads	Yes	21, 22
DeepVariant v1.2.0, 1.3.0	Convolutional neural network model trained on mapped reads	No	24
DiscoSNP v2.2.10	DeBruijn graph from k-mers of raw reads	No	33
FreeBayes v1.3.5	Haplotype inference based on mapped reads	Yes	23
GATK HaplotypeCaller v4.0.11.0	Local reassembly of mapped reads to generate potential haplotypes, followed by reads realignment to candidate haplotypes.	Yes	25 – 27
Lancet v1.1.0	Mapped reads decomposed into deBruijn graph of k-mers	No	32
Octopus v0.7.4	User choice of combination of read pileups, local reassembly of mapped reads, realignment of misaligned repeat regions, and/or user-specified alleles, to identify candidate haplotypes.	Yes	31
VarDict-Java v1.8.2	Mapped reads with different local realignment strategies for short vs longer indels, and for complex variants comprising multiple indels.	No	30

## MATERIALS AND METHODS

### Variant simulation.

A locally developed Python script, variants.py v1.0, was used to introduce SNPs, insertions, and/or deletions into the whole-genome sequences of M. tuberculosis H37Rv (GenBank accession NC_000962.3), K. pneumoniae HS11286 (GenBank accession NC_016845.1), and S. aureus NCTC 8325 (GenBank accession NC_007795.1) to generate novel variant genomes *in silico*. variants.py adds mutations to a reference genome based on user-provided parameters for proportion of SNPs to insertions and deletions, minimum and maximum indel lengths, and mutation density. We set variants.py to evenly distribute mutations in all our simulations; this setting can be edited to produce a normal distribution of mutations instead.

We generated multiple replicates of synthetic variant genomes comprising SNPs only, insertions only, or deletions only to test the performance of variant callers on each type of mutation. In order to obtain adequate numbers of mutations for statistical inference with a low probability of introducing adjacent mutations, we performed 60 replicates with a mutation density of 1 mutation every 200 bases. This mutation density was intentionally selected to ensure a minimum of 1 mutation per read, as a way to stress-test the variant callers. Because M. tuberculosis has a genome size of 4,411,532 bp, this gave us approximately 20,000 mutations per variant genome. With a genome size of 5,333,942 bp, mutated K. pneumoniae genomes had approximately 26,000 mutations each, while approximately 14,000 mutations were introduced in each S. aureus variant genome (2,821,361 bp). Indel length was allowed to range between 1 and 50 bases, resulting in median indel length of 25 bases (mean 25.5, SD 14.4).

We also generated synthetic variant genomes from the M. tuberculosis reference strain that comprised SNPs only, insertions only, or deletions only, this time at a mutation density of 1 every 1,000 bases (approximately 4,000 mutations per mutated M. tuberculosis genome) to model genetic distance in the same order of magnitude as those between M. tuberculosis complex (MTBC) strains, which have average nucleotide identities of greater than 99% (7).

Validation of introduced mutations was done by aligning reference and selected variant genomes (one mutated genome per mutation type per species) in Mauve v2015-02-25 (8), followed by manually inspecting the sequences at 10 of the mutated coordinates, resulting in 90 mutations examined.

### Read simulation, processing, and mapping.

Synthetic paired-end reads were generated from the H37Rv reference genome and simulated variant genomes with ART v2016-06-05, with read lengths set at 150 bp (mean fragment length was 200 bp, SD 10 bp). The average read coverage for the replicate data sets with density of 1 mutation/200 bases was set to 50×, while reads generated for the data sets with 1 mutation/1,000 bases had average coverage of 100×. The latter data sets were then subsampled to average coverages of 90×, 80×, 70×, 60×, 50×, 40×, 30×, 20×, 15×, 10×, and 5× to investigate the effect of read depth on variant calling. Instead of the default base quality profile model, we provided our own, based on in-house sequencing of M. marinum, K. pneumoniae, and S. aureus. The synthetic reads were trimmed with cutadapt v1.15 (34), retaining those with minimum read quality of 5 and minimum length of 20 bp. We also used cutadapt to detect and trim Illumina adaptor sequences.

Preprocessed reads were then mapped to the H37Rv reference genome with the “bwa mem” v0.7.13 (35) algorithm. The resulting BAM files were coordinate-sorted and indexed with samtools v1.2 (18, 19). Duplicated reads and poorly mapping reads (MAPQ < 10) were removed using GATK MarkDuplicates (Picard) v4.0.11.0 and “samtools view,” respectively.

### Variant calling.

The final BAM files were used as input for seven different variant callers: bcftools, DeepVariant, FreeBayes, GATK HaplotypeCaller, Lancet, Octopus, and VarDict. We also tested an eighth variant caller, DiscoSNP, which is a reference-free method that does not use BAM files as input; instead, DiscoSNP uses raw read sets in kmer-based deBruijn graph analysis to identify variants. While the somatic variant caller Lancet also uses kmer-based deBruijn graphs, Lancet decomposes mapped reads from BAM files. In order to take advantage of Lancet’s joint analysis of tumor and normal samples, we simulated and mapped reads from the H37Rv reference genome to use as the normal reference reads; see *Read Simulation, Processing, and Mapping* above.

Where the options were available, ploidy was set to one (bcftools, FreeBayes, GATK, Octopus), and minimum alternate allele fraction set to 0.2 (FreeBayes, VarDict, Lancet) to reflect expected variant frequencies in haploid genomes. Other than ploidy and alternate allele fraction, default parameters were used in all variant callers; we assumed the multiallelic variant-calling model as the default setting for “bcftools call.”

### Variant normalization and VCF filtering.

Since each variant caller can have different default information reported in their output VCFs, we postprocessed all VCFs so that variant sequences and genomic coordinates could be accurately compared to determine true/false calls for calculation of precision and recall. True positive variant calls are those with the same sequence and genomic coordinates as simulated mutations. Variant calls that differ from the simulated mutations in sequence or coordinates are considered false positive, while false-negative calls are simulated mutations that are not identified by the variant caller. Precision (or positive predictive value) is then calculated as the ratio of total true positive calls to the sum of true positive and false-positive calls, while recall (sensitivity) is the ratio of total true positive calls to the sum of true positive and false-negative calls.

Variant normalization refers to both left-aligning the variant coordinates with respect to the reference genome, and parsimoniously representing the variant sequence. GATK LeftAlignAndTrimVariants was used to normalize variant representations in all VCF files. Because most variant callers report diploid genotypes, we decided that heterozygous alternate alleles should be counted as valid variant calls as well, and only filtered out homozygous reference alleles (e.g., GT = 0/0) from VCFs that reported them.

### Data availability.

All pipeline associated code are publicly available at https://github.com/molmicdx/mtb-pipeline. The simulated synthetic genomes used for this analysis, and a Jupyter notebook containing data analysis code to reproduce these results are provided on Zenodo (doi:10.5281/zenodo.8030188).

## RESULTS

Sixty mutated genomes per mutation type (i.e., SNPs-only, insertions-only, deletions-only) were generated *in silico* from each of the three bacterial references, at a density of 1 in 200 bases and average 50× read depth, resulting in approximately 1.2 million pooled nonduplicate mutations per mutation type in M. tuberculosis, approximately 1.4 million in K. pneumoniae, and approximately 800,000 in S. aureus. Using this approach, we were able to generate a large number of observations without over-saturating individual genome locations.

**FIG 1:**
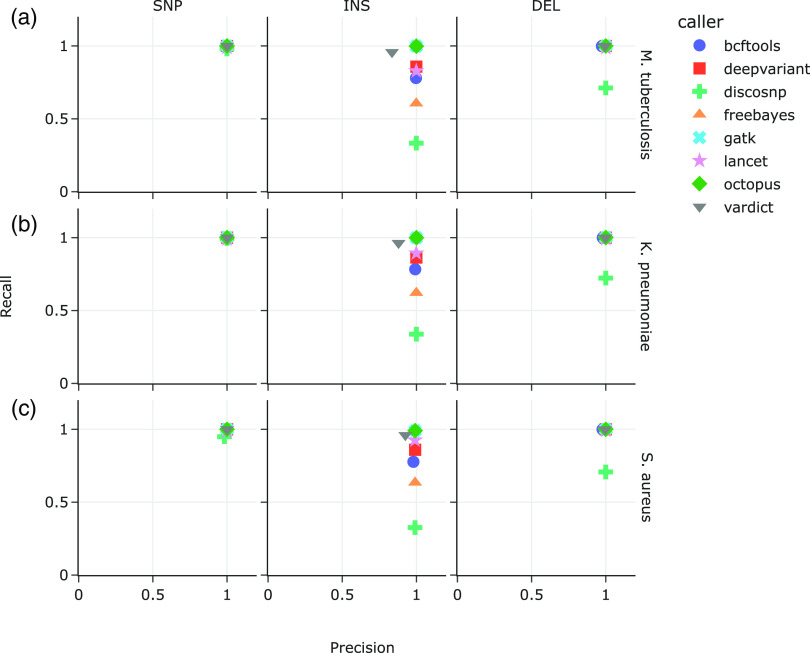
Recall versus precision on synthetic data sets with SNP-only (left), INS-only (middle), and DEL-only mutations (right). Each data set comprised 60 mutated (a) M. tuberculosis, (b) K. pneumoniae, and (c) S. aureus reference genomes. Mutations were introduced at a density of 1 for every 200 bases. Synthetic reads were generated at 50× average read depth.

All eight variant callers performed similarly well in calling variants from the M. tuberculosis data sets that comprised only SNPs (Fig. 1a), with recall ranging from 97.9% (DiscoSNP) to 99.9% (bcftools, DeepVariant, FreeBayes, GATK, Octopus, and VarDict), and precision 99.96% (DiscoSNP) to 100% (bcftools, FreeBayes, GATK, VarDict). Performance predictably declined when calling indels, with the most variable performance on the insertions-only data set (Fig. 1a, middle and right panels). Recall on the insertions-only data set ranged from 33.30% (DiscoSNP) to 99.86% (GATK), while recall ranged from 71.20% (DiscoSNP) to 99.99% (GATK) on the data sets that consisted of only deletions. Meanwhile, precision on the insertions-only data set ranged from 83.54% (VarDict) to 99.97% (GATK), and 97.39% (bcftools) to 99.99% (GATK and Lancet), on the deletions-only data set.

Similarly in the K. pneumoniae data sets (Fig. 1b), SNP recall and precision were 99.99% or better, while in the S. aureus data sets (Fig. 1c) SNP recall ranged from 94.9% (DiscoSNP) to 100% (FreeBayes), with precision ranging from 98.3% (DiscoSNP) to 99.99% (all other callers). Recall of insertions set the variant callers apart, ranging from 33.79% (DiscoSNP) to 99.93% (Octopus) in the K. pneumoniae data sets and 32.59% (DiscoSNP) to 99.09% (GATK and Octopus) in the S. aureus data sets. Precision in calling insertions meanwhile ranged from 87.97% (VarDict) to 99.99% (DeepVariant) in the K. pneumoniae data sets, and 92.54% (VarDict) to 99.14% (DeepVariant and Octopus) in the S. aureus data sets. DiscoSNP had the lowest recall of deletions in both the *K pneumoniae* and S. aureus data sets (72.26% and 70.77%, respectively), while DeepVariant, GATK, and Octopus each recalled 99.99% in both bacterial data sets. Precision in calling deletions ranged from 97.99% (bcftools) to 99.99% (DeepVariant, FreeBayes, GATK, Lancet, Octopus) in the K. pneumoniae data sets, and 97.83% (bcftools) to 100% (GATK) in the S. aureus data sets.

**FIG 2:**
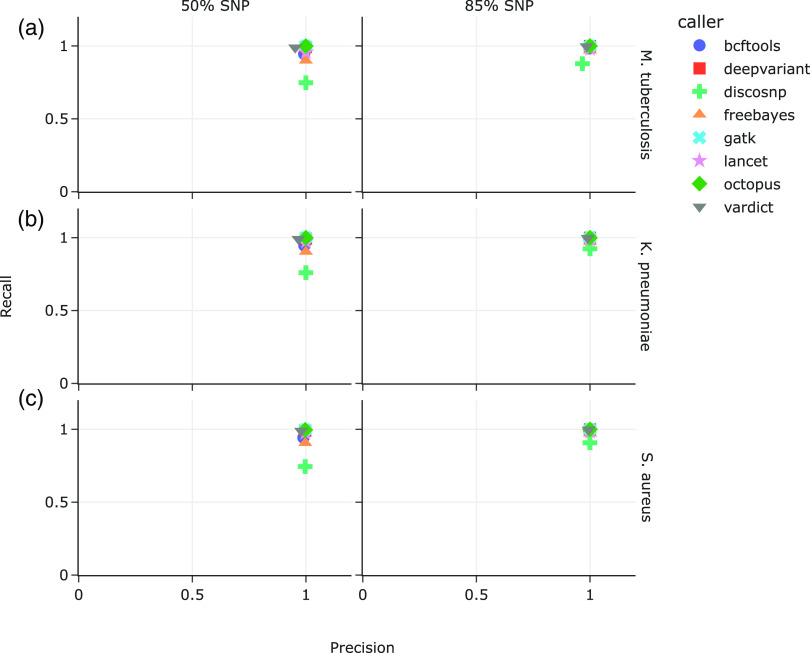
Precision versus recall on synthetic data sets with mixed variants in mutated (a) M. tuberculosis, (b) K. pneumoniae, and (c) S. aureus genomes. Left panel: 50% SNPs, 25% insertions, 25% deletions; right panel: 85% SNPs, 7.5% insertions, 7.5% deletions.

We also analyzed data sets consisting of mixed mutation types, similarly introduced at a density of one every 200 bases (approximately 1.2 million, 1.4 million, and 800,000 mutations per M. tuberculosis, K. pneumoniae, and S. aureus data sets, respectively). The first data set comprised 50% SNPs and 25% each of insertions and deletions, while the second data set consisted of 85% SNPs and 7.5% each of insertions and deletions (Fig. 2); insertions in both data sets comprised 50% duplications, and 50% inversions or random sequences. Median indel length was 25 bp (mean 25.5, SD 14.4).

In the M. tuberculosis data sets, recall and precision when SNPs comprised 50% of the mutations ranged from 74.79% (DiscoSNP) to 99.95% (GATK and Octopus), and 95.26% (VarDict) to 99.97% (FreeBayes, GATK, Lancet, and Octopus), respectively. Recall and precision improved when the ratio of SNPs was increased to 85% in M. tuberculosis, ranging from 87.82% (DiscoSNP) to 99.98% (GATK and Octopus), and 96.67% (DiscoSNP) to 99.99% (DeepVariant, FreeBayes, GATK, Lancet, and Octopus), respectively (Fig. 2a). This pattern is similarly observed for K. pneumoniae (Fig. 1b) and S. aureus (Fig. 1c). Recall and precision for K. pneumoniae with 50% SNPs ranged from 76.0% (DiscoSNP) to 99.98% (GATK), and 96.7% (VarDict) to 99.99% (FreeBayes and GATK), respectively. At 85% SNPs, recall in K. pneumoniae ranged from 92.4% (DiscoSNP) to 99.99% (GATK and Octopus), with > 98% precision for all variant callers. Recall and precision for S. aureus with 50% SNPs ranged from 74.5% (DiscoSNP) to 99.7% (GATK and Octopus), and 97.9% (VarDict) to 99.70% (FreeBayes and GATK), respectively. Finally, recall for 85% SNPs in S. aureus was 90.8% (DiscoSNP) to 99.99% (GATK), with > 99% precision for all variant callers.

We then partitioned the indels simulated in Fig. 1 (middle and right panels for all reference genomes, i.e., 120 mutated genomes per species) by size to determine the effect of indel length on the variant callers’ ability to recall them. The total number of insertions and deletions 21 to 50 bp in length varied based on reference genome size, ranging from 946,920 (S. aureus) to 1.8 million (K. pneumoniae). Indels 6 to 20 bp in length ranged from 474,617 to 894,070 while indels 1 to 5 bp in length ranged from 157,691 to 297,351.

**FIG 3:**
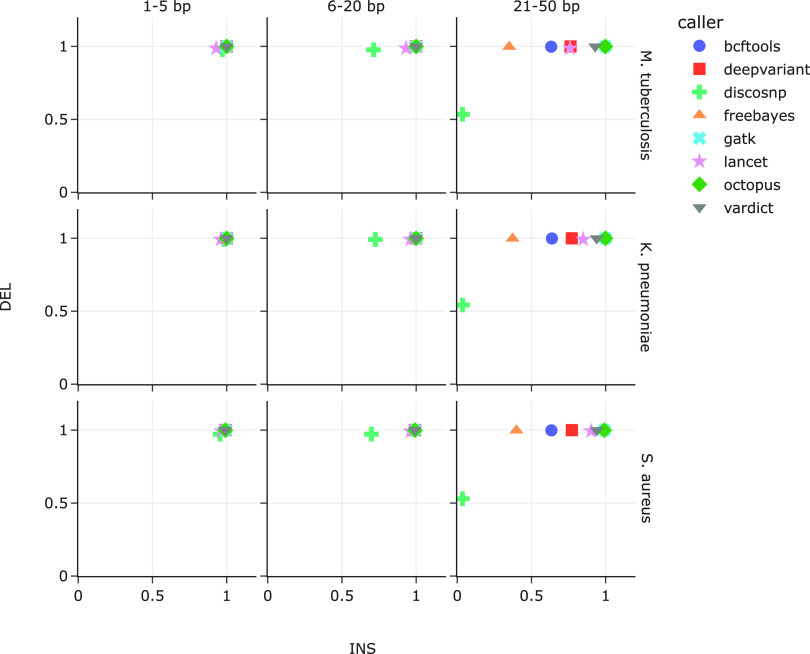
Recall of introduced deletions versus insertions by each variant caller. Indels were introduced into M. tuberculosis (top row), K. pneumoniae (middle row), and S. aureus (bottom row), and are binned by size: 1 to 5 bp (left), 6 to 20 bp (middle), and 21 to 50 bp (right).

The most consistent pattern differentiating variant caller recall in all three bacterial references is the length of insertions, while deletion length did not significantly alter recall (Fig. 3). Across all three bacterial reference genomes, insertions 21 to 50 bp in length significantly differentiated the variant callers, with recall ranging between 3.5% to 3.6% (DiscoSNP) and 99.0% to 99.9% (GATK and Octopus). The recall range was much narrower for shorter insertions. Recall of insertions 6 to 20 bp in length ranged between 69.9% to 72.5% (DiscoSNP) and 99.15% to 99.98% (VarDict), while recall of insertions of sizes 1 to 5 bp ranged between 92.9% to 96.0% (DiscoSNP and Lancet) and 99.2% to 99.99% (bcftools and VarDict). Unlike insertions, deletions of varying lengths did not appear to affect variant caller as strongly, with recall values over 97% across all lengths and reference genomes. The exception was DiscoSNP; while DiscoSNP’s recall of 6 to 20 bp deletions was also above 97%, this deteriorated to 54% with deletions 21 to 50 bp in length.

**FIG 4:**
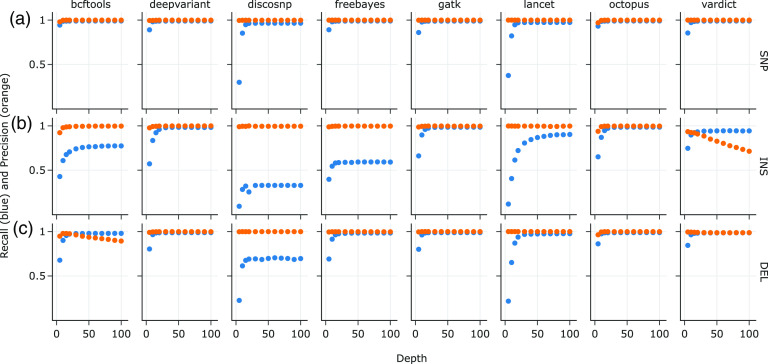
Recall and precision on synthetic M. tuberculosis data sets with 1 mutation/1,000 bases per simulation to approximate the genetic distances between M. tuberculosis complex (MTBC) species, at varying read depths for the (a) SNP-only, (b) INS-only, and (c) DEL-only data sets.

Finally, we also investigated the effects of varying read depths from 5× up to 100× at a mutation density of one for every 1,000 bases on variant caller performance, focusing on the M. tuberculosis mutated genomes, because recall and precision trends in all mutation types are recapitulated in both K. pneumoniae and S. aureus. At a density of 1 mutation every 1,000 bases, the total mutation counts were 4,398 for the SNPs-only data set (Fig. 4a); 4,315 for the insertions-only data set (Fig. 4b); and 4,261 for the deletions-only data set (Fig. 4c). Across all variant callers, recall and precision in the SNPs-only data set were ≥ 95% and ≥ 99%, respectively, at read depths of 20× and higher (Fig. 4a).

For the insertions-only data set, however, recall at 20× ranged from 25.5% (DiscoSNP) to 97.5% (GATK) (Fig. 4b). The influence of read depth on recall also varied widely by variant caller, with the point of rapid decline in performance at 70× coverage for Lancet (89% recall) and lowest of 15× for FreeBayes (58% recall) (Fig. 4b). Precision of the variant callers on the insertions-only data set at 20× was similar to the SNPs-only data set at ≥ 99%, except for VarDict at 90.8%. Interestingly, VarDict’s precision was inversely correlated to read depth, steadily decreasing from 93% at 5× to 71% at 100×.

All of the variant callers performed slightly better on the deletions-only data set than on the insertions-only data set at 20× read depth (Fig. 4c), with recall ranging from 68.9% (DiscoSNP) to 98.7% (GATK) and precision ranging from 97.55% (DiscoSNP) to 99.98% (GATK and Lancet). VarDict’s precision only declined slightly between 5× and 30× to 98% on the deletions-only data set; however, precision of bcftools dropped from 97.9% to 89.3% between 10× and 100×.

**FIG 5:**
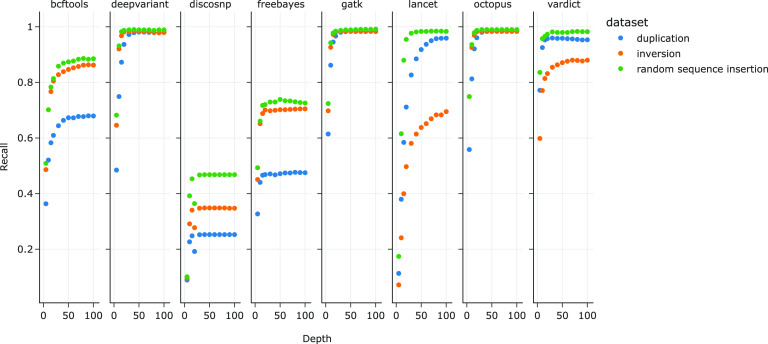
Recall values for insertions consisting of duplications, inversions, and random sequences. Precision was not calculated since false-positive calls did not have information on the type of insertions that were identified by the variant callers.

Three types of insertions were introduced into the insertions-only data set referenced in Fig. 4b: duplications, inversions, and random sequences, at a ratio of 2:1:1. In order to further investigate the features of insertions that determine recall for each variant caller, we subdivided the same insertions-only data set by the types of insertions introduced (Fig. 5): duplications-only (2,220 total mutations), inversions-only (1,016), and random sequences only (1,079). Median insertion lengths were 26 bp (mean 25.7, SD 14.4), 25 bp (mean 25.6, SD 14.3), and 24 bp (mean 24.8, SD 14.6) for duplications, inversions, and random sequences, respectively. At read depths of 20× and higher, recall did not significantly differ between duplications, inversions, and random sequences for DeepVariant (respectively 93.6%, 98.1%, and 98.7%, at 20×) and GATK (96.7%, 98.1%, and 98.4%). However, recall of duplications were the lowest of the three insertion types for DiscoSNP (max. 25.3% at ≥ 40×), FreeBayes (max. 47.7% at 40×), and bcftools (max. 67.9% at 90×), while recall of inversions were lowest for Lancet (max. 69.5% at 100×) and VarDict (max. 87.9% at 70× and 100×).

## DISCUSSION

Identifying single nucleotide polymorphisms from whole-genome sequencing short reads has become a relatively straightforward task that most popular variant calling software and pipelines can reliably perform, especially with high-quality sample preparation and trusted reference databases. More recent advancements in identifying SNPs focus on either calling with limited signal information (e.g., low-frequency variants), or reducing computational burden. Accurately identifying indels and structural variants, however, remains a challenge. Developing effective algorithms for calling complex variants is one part of the challenge, while the other is generating a reference set of known variants to validate against. This study was specifically motivated by the need for a principled approach for selecting a variant caller and defining relevant parameters in the process of developing and validating a clinical assay for predicting antimicrobial resistance in M. tuberculosis. Additional species were included to ensure that this approach is generalizable to other genomic contexts.

Application of whole-genome sequencing in the clinical microbiology laboratory can aid clinicians in predicting antimicrobial resistance (9). Markers of antimicrobial resistance include insertions, deletions, and other complex mutations in addition to SNPs, necessitating the use of variant callers that are robust in detecting such mutations. For example, indels were found to be enriched in the antibiotic resistance-conferring genes of M. tuberculosis strains involved in the Central Asian multidrug-resistant outbreak (10). Small indels in M. tuberculosis were also associated with increased MIC of the antibiotics levofloxacin, ethionamide, and delamanid (11). Resistance traits associated with indels and other complex mutations are also significant in other species. Integration of the SCCmec cassette in the chromosome of S. aureus, for example, confers methicillin resistance (12), which can also subsequently be inactivated through insertion of Tn551 (13). Polymyxin resistance is also associated with deletions in the PhoPQ gene in K. pneumoniae and other bacterial species (14).

Methods for indel detection generally employ gapped alignment, split reads, *de novo* assembly, or a combination thereof (15). Gapped alignment methods detect small indels that are contained within the length of a read; split-read methods identify medium-length indels (10 bp to 1 kb) at the cost of missing low-frequency indels, while *de novo* assembly methods can identify larger indels, albeit by utilizing significant computational resources (15). Given that identifying variants is an integral part of clinical genomics, variant callers and other software used as part of the bioinformatics workflow are often validated and benchmarked against human reference data sets (e.g., 6, 15, 16). Variant calling in the context of resolving clinical infections or tracking infectious disease transmission and epidemiology also relies on the same bioinformatics tools; therefore, these tools also need to be tested against microbial data sets.

Recent efforts to validate widely used bioinformatics tools with microbial data include testing SNP-calling assemblies and pipelines against increasingly divergent bacterial reference genomes (1, 4), and combining whole-genome sequencing and traditional molecular biology assays to validate a SNP-calling bioinformatics workflow (17). Steglich and Nübel (18) tested four indel callers against 1- to 2,321-bp indels introduced into a reference Clostridium difficile bacterial genome *in silico*, and found that while gapped alignment (e.g., as implemented in FreeBayes) was suitable for indels ≤ 29 bp, a combination of methods as employed by the ScanIndel framework (15) was best for indels > 29 bp.

In the present study, we synthetically generated variant whole-genomes based on three bacterial reference genomes to test the performance of eight variant callers that are designed to identify both SNPs and short indels. Within the context of a clinical workflow, variants are primarily identified by comparing isolates against reference strains that are medically informative for treatment or epidemiological tracking. For example, the WHO’s catalog of mutations associated with antimicrobial resistance in M. tuberculosis are identified with reference to the H37Rv strain of the pathogen (19). We therefore used the same M. tuberculosis H37Rv reference genome for our analyses, as well as K. pneumoniae HS11286 and S. aureus NCTC 8325 reference genomes for the respective data sets. Selection of an appropriate reference genome has been shown to be the main factor affecting SNP calling sensitivity and precision, with decreasing pipeline performance as genetic distance between reads and reference genome increased (1). Our results correspond with Bush et al.’s findings (1); variant-calling performance generally suffered with increased ratio of indels to SNPs (Fig. 2), and increased indel lengths (Fig. 3).

The three selected reference strains are varied in genome size and GC content, ranging from S. aureus with a genome size of 2.8 Mbp and %GC 32.9 to K. pneumoniae with the largest genome (5.3 Mbp) and M. tuberculosis with the highest %GC (65.6 Mbp). The differences in reference genome sizes and GC content did not appear to affect variant caller recall and precision as they performed similarly across all three bacterial species. Genomic regions with homopolymers and repetitive sequences are known to introduce ambiguous read mapping results; these regions can be masked to reduce the amount of false-positive calls (2). This is typically done for example, in the highly repetitive PE/PPE regions of the M. tuberculosis genome (17, 20). We did not restrict variant simulation or calling to any loci in this study; simulated variants were evenly distributed across the genomes. Our results indicate that masking these regions was generally unnecessary for these variant callers to accurately identify SNPs alone. Further work is needed to examine the relationship between indel length, genome complexity, and variant caller performance.

The widely used “bcftools mpileup” and “bcftools call,” which evolved from “samtools mpileu” and “bcftools view,” applies a multiallelic variant-calling model to identify SNPs and indels by statistically inferring allele and haplotype frequencies based on “pileups” of reads aligned to a reference genome (21, 22). FreeBayes also models multiallelic loci within a Bayesian statistical framework based on aligned reads in order to detect variation across inferred haplotypes (23). DeepVariant meanwhile, takes advantage of images of read pileups and known true diploid genotypes to train a convolutional neural network model to subsequently identify new candidate SNP and indel variants (24).

Instead of strictly utilizing genomic position-based pileups, GATK HaplotypeCaller uses a consensus of reads at genomic regions of interest to assemble theoretical haplotypes from deBruijn-like graphs (25–27). VarDict similarly relies on aligned reads, then uses soft-clipped reads (reads with mismatched bases that are masked for the purposes of alignment but not permanently trimmed) (28, 29) for local realignment to estimate indel allele frequencies (30). The variant caller Octopus, also uses k-mer-based deBruijn graphs for local reassembly, in combination with read pileups and common patterns of misalignments to identify alleles and generate candidate haplotypes (31). The somatic variant caller Lancet also locally assembles mapped reads, then decomposes them into k-mers to construct deBruijn graphs that are color-labeled to differentiate tumor versus normal samples (32). DiscoSNP meanwhile eschews aligned reads completely, instead constructing deBruijn graphs of k-mers from raw read sets to detect SNPs and indels (33).

While all tested variant callers generally excelled at identifying SNPs, the variant callers that were best at recalling insertions in the three bacterial reference genomes, GATK HaplotypeCaller, Octopus, and VarDict (Fig. 1), all scrutinize genomic regions with discrepancies such as base mismatches and high quality soft-clipped bases to identify indels (27, 30, 31). This also suggests an explanation for DiscoSNP’s poor recall because local alignment context is lost when only k-mers of raw reads are used for variant identification. GATK HaplotypeCaller and VarDict’s high recall corresponds with the performance of the ScanIndel framework; the latter also recovers soft-clipped reads for realignment before final variant calling, which helps to identify medium-sized insertions and large deletions (15). Interestingly, higher coverage depth up to 100× and lower mutation density caused VarDict to falsely call more insertions than GATK HaplotypeCaller did (Fig. 4b), by up to 3 orders of magnitude. Lai et al. (30) describes VarDict's strength in detecting deletions and complex variants that include deletions, which are supported by our observations in the deletions-only data set. The deletions-only data set also posed less of a challenge to the other tested variant callers, presumably because deletion breakpoints are simpler to identify by the lack of reads or read segments.

We initially expected variant callers that allow specific parameterizations to reflect haploid genomes to perform better due to more accurate expectations of variant allele frequencies or genotypes. The ability to parameterize ploidy (Table 1) did not appear to have a significant effect on recall of insertions. Of the variant callers where ploidy could be set to 1, bcftools and FreeBayes consistently had poorer recall of insertions than GATK HaplotypeCaller and Octopus. On the other hand, DeepVariant and Lancet, which do not have ploidy settings, demonstrated better recall of insertions than bcftools and FreeBayes did (Fig. 1). Specifying the minimum fraction of variant alleles to reflect expected haploid variant frequencies may have helped in the case of Lancet, but not FreeBayes. Nevertheless, we note that failing to set the variant allele fractions in callers that had this parameter available resulted in excessive amounts of false-positive calls.

The variant callers surveyed in this study employ different strategies that ultimately attempt to deal with the problem of aligning reads that do not match the reference sequence. Our data indicate that algorithms that take advantage of local genomic context, especially when there are significantly mismatched or soft-clipped bases, perform better than those that do not; however, the specific local realignment strategies adopted by the variant callers matter in differentiating their respective performances on insertions versus deletions. Ultimately, the selection of a variant caller for use in laboratory-developed tests should be informed not only by performance characteristics, but also by the quality of documentation, organizational familiarity with computing languages and platforms, whether the software project is actively maintained, and usage patterns in the literature and laboratory community.
